# Nursing Students’ Practicums during the COVID-19 Crisis and the Effect on Infection-Prevention Behavior in Students: A Mixed-Method Approach

**DOI:** 10.3390/medicina57121354

**Published:** 2021-12-12

**Authors:** Hiromi Nakagawa, Hiroyuki Sasai

**Affiliations:** 1 School of Nursing, Takarazuka University, 1-13-16 Shibata, Kita-ku, Osaka 530-0012, Japan; 2 Research Team for Promoting Independence and Mental Health, Tokyo Metropolitan Institute of Gerontology, 35-2 Sakae-Cho, Itabashi-ku, Tokyo 173-0015, Japan; sasai@tmig.or.jp

**Keywords:** COVID-19, coronavirus, nursing practicum, infection prevention, behavior change

## Abstract

*Background and Objectives:* The coronavirus disease pandemic is ongoing. Infection-prevention measures in nursing education (practicum) are essential. However, there are few studies on infection-prevention behaviors among nursing students participating in practicums. We aimed to clarify the effect of practicums during the coronavirus disease crisis on infection-prevention behavior in Japanese nursing students. *Materials and Methods*: We conducted semi-structured interviews with 13 third-year nursing students in Osaka City within one week of their clinical placement training. From the results of the interview analysis, we compiled a questionnaire and surveyed 90 third-year students. We conducted qualitative and quantitative analyses. We used descriptive statistics for the quantitative analysis and the chi-squared test for binary variables. *Results:* From the qualitative analysis, we identified five categories regarding the awareness of infection-prevention measures: <Acquisition of knowledge and skills in infection-prevention measures during nursing practice>, <Defining the experience in infection-prevention measures>, <Changes in attitude towards infection>, <Changes in infection prevention behavior>, and <infection-prevention measures-related issues>. In the quantitative analysis, the practicum students who attended at least three pre-practicum orientations continued wearing masks during lunch breaks and avoided the three Cs. *Conclusions:* Students could recall the knowledge and experiences gained from pre-practicum orientations/practicums. This experience created a new awareness of infection-prevention and change of infection-prevention behavior. Infection-prevention education using practicums is important for infection-prevention behavior during this pandemic. However, there should be a much larger-scale study to support these findings in the future.

## 1. Introduction

Coronavirus disease (COVID-19) is an infection caused by the severe acute respiratory syndrome coronavirus 2 (SARS-CoV-2). In approximately 80% of patients with COVID-19, the disease is asymptomatic or mild. However, the disease can be fatal when a cytokine storm causes acute respiratory distress syndrome. The incubation period of SARS-CoV-2 goes up to 14 days, and the infection usually develops within five days of exposure [1,2]. As of September 2021, there were 228,807,631 cases and 4,697,099 deaths worldwide [3], and 1,681,120 cases and 17,269 deaths in Japan [4]. We expect further increases, given the transmission of variants around the world. Concomitantly, there are visible long-term effects on nursing education due to the clusters developing in medical and nursing care facilities and the pressure placed on the medical profession resulting in the suspension of clinical placement practicums.

A survey of 263 member universities and two academies conducted by the Japan Association of Nursing Programs in Universities on the implementation of clinical placement practicum courses (compulsory) for fourth-year university nursing students reported that only 13 out of 695 (1.9%) planned practicum courses were conducted [5]. The proportion at our university was 32.5% of the scheduled clinical placement practicums. Consequently, to guarantee learning outcomes, our university used hybrid practicum courses with a combination of online practicums and on-campus in-person practicums. The merit of clinical placement practicums is skill acquisition that enhances the practical nursing ability and clinical judgment. Nevertheless, there is an urgent need for infection-prevention measures. A previous online survey assessed 99 Australian and 113 Indian nursing students on COVID-19 preventive behaviors. The study discussed the suitability of the “new normal” of online learning and other issues [6]. However, we did not find studies conducted in Japan or elsewhere on COVID-19 infection-prevention measures in clinical placement practicums. Therefore, we aimed to clarify the effect of practicums (clinical placement practicums and on-campus practicums) on infection-prevention behavior (IPB) in nursing students during the COVID-19 crisis. This study used both qualitative and quantitative analyses. We hypothesized that “nursing student practicums during the COVID-19 crisis affect infection-prevention measures in students”. The findings from this study will help develop a pre-practicum educational program for infection-prevention measures (IPMs) for nursing students.

## 2. Materials and Methods

### 2.1. Study Design, Setting, and Participants

We used a mixed-method approach in our study design. The participants were third-year students from a single Japanese nursing university in the 2020 academic year. The inclusion criteria were students who had completed all field-specific practicums, including clinical placement practicums and on-campus practicums, and students who gave their consent for participation. Additionally, the study involved only participants who took the 5-hour practicum guidance course, including nursing skills practice. The participants took didactic courses “Infection and Immunity” and “Biology” in their first year of college, and all successfully received credits for them.

They also learned and obtained credits for handwashing, personal protective equipment (PPE), and standard precautions in the introductory nursing courses. The exclusion criteria were those with incomplete practicums or who did not provide consent for the study. We recruited third-year students because orientation content and subject acquisition status, including locations of practicums and infection-prevention education, differed between years after the state of emergency declaration. None of the participants had COVID-19 before or after the nursing practicum. The study period was 28 September 2020 to 30 June 2021, and the location was a nursing university in Osaka City.

#### Operational Definition of Terms

In this study, “practicum” referred to clinical placement practicums and on-campus practicums. “Awareness” meant being conscious of something and understanding its meaning. “Behavior change” [7] meant changing long-term behaviors through experience and a learning process.

### 2.2. Procedure

From the results of the qualitative analysis, we compiled a survey questionnaire to investigate the participants’ awareness of IPMs, after which we conducted a quantitative study.

#### 2.2.1. Qualitative Research

The participants of the qualitative analysis were 13 third-year students who had completed practicums in adult nursing, gerontological nursing, women’s health nursing, community health nursing, child health nursing, and psychiatric and mental health nursing. It was impractical to verify any differences in training since all the participants received this training beforehand. We conducted the interviews within a week of completion of the practicum.

We randomly selected participants using a random number table. We used a semi-structured interview method for data collection.

##### Data Collection Procedure

All the nursing students attended a pre-practice orientation session once. In addition, there was a pre-practice orientation for each practicum. Participants attended a total of seven sessions. We delivered a 90-minute lecture on handwashing, hand sanitizing, and skills practice relating to PPE as orientation for infection control measures before on-campus practicums and clinical placement practicums. We explained the study to students who completed their practicum within the specified period. We used a study cooperation request form to recruit participants. The participants gave consent and met the inclusion criteria. We conducted interviews with the approval of the Takarazuka University Research Ethics Review Committee. Then, we conducted semi-structured 45-minute interviews in a private room with participants who completed their nursing practice after receiving pre-practicum orientation.

##### Interview Content

We asked the participants about (1) their feelings before and after the practicum; (2) IPMs before, during, and after the practicums; (3) challenging aspects of IPMs; (4) health/behavior management checks after the practicums; (5) action they had to take if they or another class member developed a fever; and (6) attitude toward IPMs. We obtained consent from the participants for the interview content, and we recorded the interviews with a voice recorder.

#### 2.2.2. Quantitative Research

We found in the qualitative analysis that student awareness of IPMs in practicums during the COVID-19 crisis affected IPB after the practicum. Then, we hypothesized that student awareness came from pre-practicum orientation, and the experience during the practicums, which changed the students’ IPB. Therefore, we based the conceptual framework on the students’ ability to recall the knowledge and experiences gained from the pre-practicum orientation and the practicums. Thus, this process created a new awareness about infection prevention, resulting in a change in IPB.

In the quantitative analysis, the object variable was IPB. The dependent variable was awareness of IPMs. Additionally, we investigated the factors affecting post-practicum changes in IPB, including the number of orientations and practicum experiences and the anxiety and feelings of difficulty before and after the practicums. As extracted from the qualitative analysis, student awareness of IPMs in practicums during the COVID-19 crisis affected IPB after the practicum. Then, we hypothesized that students became aware of IPMs from the pre-practicum orientation and practicums, changing their IPB. Therefore, the conceptual framework was based on students’ ability to recall the knowledge and experiences gained from the pre-practicum orientation and practicums. This process created a new awareness of infection prevention, which resulted in a change in IPB.

On 23 June 2021, we organized a questionnaire-based survey at the university. The principal investigator provided sufficient information about the study, after which the participants provided informed consent. We considered that responding to the questionnaires was consent for participation. We explained that it would be impossible to ensure anonymity with consent withdrawal. Instead, we distributed the questionnaire forms together with envelopes. It was up to the participants (and without coercion from the researchers) to place the anonymous completed questionnaire in the envelope and then into a locked mailbox in the university’s secretariat. The collection period was one week from the day of the questionnaire distribution.

The principal investigator collected and managed the questionnaires in the research laboratory.

##### Items in the Questionnaire-Based Survey

(1) Participant attributes: age, gender, number of clinical placement practicums, and health status.
(2) Motivations: Factors related to anxiety before and after preparation for practicums.
(3) Acquisition of knowledge and skills: Subjects studied and practicum orientation topics that were helpful for IPMs during the practicums.
(4) Nursing practice: IPMs learned during practicums, IPMs used by students, and difficulties faced during practicums.

### 2.3. Statistical Analysis

#### 2.3.1. Qualitative Research

We transcribed the responses as verbatim records, and the interpreted definitions were the descriptions expressed without any loss of context. Next, we performed an initial coding on the original data. Then, we generalized the content of the analysis units to the abstract level and generated coded units to express the content. Finally, we combined the extracted and coded units into subcategories and integrated the characteristics of meaning in each context unit to create inductive categories and subsequently assigned labels.

We considered < > to signify the categories, “ for the subcategories, and ” for the codes. The research supervisor supervised the data collection and analysis to increase precision. In addition, we consulted individuals with experience in qualitative research for the content for analysis.

#### 2.3.2. Quantitative Research

First, we recorded the participants’ attributes. Next, we compared anxiety factors/learning around the practicums and changes in IPMs. Finally, we compared IPMs continued after completing practicums, according to the number of orientations and the number of clinical placement practicums. We used descriptive statistics for the statistical analysis.

The object variables were each variable, and the classification variables were before and after the practicum. We used the chi-squared test for categorical variables to determine the differences between the groups. We expressed these as mean values ± standard deviation (SD). We used SPSS Statistic ver. 27 for Windows for statistical processing, and *p* < 0.05 was the level of statistical significance.

## 3. Results

### 3.1. Qualitative Research

#### 3.1.1. Overview of the Participants

Participants comprised nine female and four male students, with an average age of 21.5 years. The total interview time was 605 min, with an average of 46.5 min per interview.

#### 3.1.2. Results of the Analysis

We created 15 subcategories from 48 codes related to nursing students’ awareness of IPMs for practicums during the COVID-19 crisis. They were aggregated into five categories as follows: <Acquisition of knowledge and skills in IPMs during nursing practice>, <Defining the experience in IPMs>, <Changes in attitude towards infection>, <Changes in IPB>, and <IPM-related issues> (Table 1).

We identified five subcategories and 14 codes in <Acquisition of knowledge and skills in IPMs during nursing practice>. The participants learned about IPM from a connection between class learning and nursing practicums. These were subcategorized as “Re-confirmation of hand hygiene methods” and “Learning about the correct usage of PPE”.

We identified two subcategories and five codes in <Defining the experience in IPMs>. The participants stated that ‘Learning the joy of nursing from patients’ reactions,’ ‘Noticing the importance of practicing patient care in clinical placement practicums,’ and ‘From nursing practicums, aiming to be a nurse that patients need.’ Experience in practicums became a “Motivation for becoming a nurse”.

We identified three subcategories and ten codes in <Changes in attitude towards infection. In “Anxiety about infection before practicums”, there was ’Student anxiety about infecting patients, medical professionals, or family.’ In contrast, there was an “expectation for learning with practicums”. At the end of the practicums, there was a change in attitude from ‘Reassurance that there were no infections among patients or medical professionals and no students who infected medical professionals’ to “Relief and a feeling of achievement after safe completion of the practicums”.

We identified two subcategories and nine codes in <Changes in IPB>. The students spoke of “Making handwashing and mask-wearing habits” and “Reducing outings and eating out times with the family” due to “Heightened awareness of health management from filling out the health/behavior management chart”. They were implementing “IPM for their family”. Additionally, the students practiced “Gathering information relating to infections from reliable information sources”, such as practicum orientations or official sources. We observed behavior changes, such as “Thinking about IPM from the perspective of daily routines”.

In <IPM-related issues>, we identified three subcategories and ten codes. For example, in “Stress due to IPM”, students spoke of aspects related to products, such as ’Hand irritation due to frequent handwashing and sanitizing’ and ’difficulty of goggle use due to fogging up or slipping off.’ In “Lack of knowledge relating to infection measures”, there was “Lack of knowledge of microbiology and infectious disease medicine” and “Lack of knowledge relating to hand sanitizers or disinfectant drugs”. Additionally, the students also experienced psychological stress such as not being able to maintain social distancing or “Not being able to get a change in scenery after practicums due to outing limitations”. Furthermore, there was “Hesitancy toward health consultations if one developed a fever” and “A feeling of guilt”.

### 3.2. Quantitative Research

We received responses from 81 participants (90.0% response rate), and 100% were valid, i.e., a minimum of nine answered questions out of ten.

#### 3.2.1. Overview of the Participants

The average age of the students was 21.3 (SD, 0.7) years (Table 2). There were 12 (14.8%) male students, 66 (81.5%) females, and three (3.7%) others. Of the seven fields of clinical placement practicums, students participated in an average of two practicums (number of fields), and the most attended was the adult nursing practicum. Six of the 90 (6.6%) third-year students reported contracting the disease outside clinical placement practicums. All students took their temperature three times a day for two weeks on either side of the practicum. They submitted a body temperature management chart.

#### 3.2.2. Learning about IIPMs from the Pre-Practicum Orientation and Previously Completed Subjects

Forty-seven participants (54.1%) attended three or more pre-practicum orientation sessions, and all students participated in at least one session. (Table 3). Seventy-seven (95.0%) participants responded that the IPMs taught during the orientation were helpful. The helpful IPMs were standard precautions (77.8%), infection routes (75.3%), and the timing of hand sanitizing (71.6%). Thirty-two (39.5%) participants acquired this knowledge during an orientation session regarding the three Cs.

#### 3.2.3. Changes in Anxiety Factors before and after Practicums

Before practicums, anxiety factors were ‘A student infecting someone else’ and ‘A student being infected by someone else’ in 54 (66.7%) participants. Additionally, the lack of knowledge of infection control was 9.9%, which dropped to 6.2% after the practicum (*p* < 0.01) (Table 4).

#### 3.2.4. A Comparison of Infection Control Measures Learned by Students from a Clinical Nurse and a Nursing Lecturer

The students learned about infection control through nursing practice from a clinical nurse. Significant infection control measures learned were correct usage of PPE (74.1%), hand hygiene (72.8%), standard precautions (64.2%), and infection risk factors (61.7%). These were significantly higher (*p* < 0.01) than those learned from a nursing lecturer (Table 5). Meanwhile, infection control measures learned from a nursing lecturer were infection prevention at lunchtime (55.6%) and shortening care time (28.4%). These had a higher statistical significance (*p* < 0.01) than those learned from the clinical nurse.

#### 3.2.5. Students’ Difficulties Surrounding IPMs

The most common difficulty of students’ surrounding IPMs was “difficulty using goggles”, followed by ”hand irritation due to hand hygiene”, “feeling down”, and ”not able to have a change in scenery”. Nineteen (23.5%) felt that “Mask wearing at lunchtime” was difficult.

#### 3.2.6. Changes in IPBs during and after the Practicum

Table 6 details the changes in IPBs during and after the practicum. Regarding changes in IPMs observed during and after the practicum, the number of IPBs decreased significantly after the practicum period (*p* < 0.05). Notably, “Mask wearing at lunchtime” reduced significantly.

#### 3.2.7. IPMs Practiced after the Practicums

Table 7 shows details of IPMs continued after the practicums according to the number of pre-practicum orientations. For students who took three or more sessions of orientation, ‘Infection prevention at lunchtime,’ ‘Avoiding the three Cs,’ and ‘Avoid outings’ were significantly higher (*p* < 0.05) than those who took less than three sessions. Furthermore, in students who participated in less than three sessions of clinical placement practicums (no. of fields), the continuation of hand hygiene was significantly higher (*p* < 0.05) than in students who participated in three or more sessions.

## 4. Discussion

The qualitative analysis suggested that student awareness of IPMs in practicums during the COVID-19 crisis changed from <Acquisition of knowledge and skills in IPMs during nursing practice> to <Defining the experience in IPMs> and then to <Changes in attitude towards infection> along with <Changes in infection prevention behavior>. However, there was psychological stress associated with IPM and hand irritation caused by hand hygiene, and thus <IPM-related issues>. Considering these results, in the quantitative analysis, the students who completed practicums after participating in at least three orientation sessions continued to wear masks during their lunch breaks and avoided the three Cs. These results show that students could recall the knowledge and experiences gained from the orientation and practicums, creating a new awareness about infection-prevention and changing IPB. Accordingly, we will consider the effect of IPM education provided through pre-practicum orientations and practicums on the IPB of students.

### 4.1. The Effect of Infection Prevention Measure Education Provided through Pre-Practicum Orientation and Practicums on the IPB of Students

This study involved students who participated in orientation sessions and then practicums. They adhered to primary prevention strategies, such as hand hygiene, mask-wearing, and avoiding the three Cs. The students changed from practicing secondary prevention measures to preventative measures after observing their temperature follow-up charts. After participating in at least three orientations, the students who completed practicums continued to wear masks during their lunch breaks and avoided the three Cs. These were IPMs the students were ignorant about pre-pandemic. Repeated participation in orientations and practice of IPMs improved the students’ awareness of IPMs.

The transtheoretical model (TTM) by Prochaska [7] is a theory of health behavior change, which comprises four hypotheses: stages of change, processes of change, self-efficacy, and decisional balance. After the pre-practice orientations and practicums, the students practiced the IPMs and were in the behavior change stage. The students understood the benefits of not being infected with COVID-19 and acquired knowledge and skills in IPMs during nursing practice. These helped them make IPB decisions. In addition, the fact that the students did not get infected or infect others and gained “Relief and a feeling of achievement after safely completing the practicums” increased their confidence and “Motivation for becoming a nurse”. Furthermore, self-efficacy also increased. These were cognitive and affective experiences in the process of IPB change that promoted behavioral change.

Contrastingly, the students who partook in three or more clinical placement practicums after participating in orientations did not continue hand hygiene after completing the practicums. This finding may be because we did not individually investigate the frequency of hand hygiene in on-campus practicums and clinical placement practicums.

Based on these results, we investigate hand hygiene frequency in an ongoing study. Furthermore, regarding infection prevention control education in COVID-19 practicums, we also need to evaluate the mask-wearing rate and compliance to the three Cs during the execution phase of the TTM so that the intervention can maintain IPM adherence.

From the results of this study, the reduction of anxiety related to ‘lack of knowledge of infection control’ arose from the students’ acquired knowledge from the orientations and experience from the practicums.

We did not determine the effect of the frequency of clinical placement practicums on IPB after completion of the practicums. Nevertheless, the students became aware of IPMs through the nursing practicums. Clinical nurses used hand hygiene and PPE correctly, which shortened the time for bedside care. Being a ‘close contact’ means remaining close to someone (less than 1 m distance) for at least 15 min without necessary prevention measures [11]. Students adhered to the guidelines and learned nursing skills, despite the hand irritation and difficulty wearing goggles. Given these factors, infection-prevention education using orientation and practicums enabled students to become aware of infection-prevention, which probably affected IPB.

### 4.2. Promoting and Inhibiting Changes in Infection Prevention Behaviors

Students learned IPMs from orientations and their experiences in practicums. Nevertheless, several IPM-related issues persisted after the completion of the practice. The following sections discuss the promotion and inhibition of changes in IPBs.

Anxiety about the infection may have promoted IPB change.

Pre-practicum anxiety was ‘threat awareness,’ where there was awareness of the risk of infection and the fear and significance of practicum cancelation or an effect on grades, which promoted IPB. Notably, ‘A student infecting someone else’ and ‘A student being infected by someone else’ were thought to have increased ‘threat awareness‘.

Van den Broucke [12] reports that the health belief model with simplified behaviors is the basis for changing people’s behavior. These behaviors include motivation, social norms, the right level of emotions, the replacement of risky behaviors, and the building of routine behavior or nudge use. The students participated in the practicums while maintaining social norms to obtain the practicum credits necessary for obtaining their nursing qualifications. Although they were concerned about the risk of infection, they avoided risky behaviors with IPMs.

Contrastingly, hand irritation is reportedly an impediment to IPB changes. However, since we did not investigate the connection between the frequency of hand hygiene in clinical placement practicums and on-campus practicums with hand irritation, we could not substantiate this. Furthermore, concerning ‘IPMs at lunchtime,’ the students implemented these during the practicum since they were monitored. However, these measures remained difficult for students, and therefore they did not change their behavior after completing the practicums. Therefore, there is still a need for enhanced education.

Based on these results, we are designing a study on the frequency of hand hygiene to assess the effectiveness of IPM education during the COVID-19 crisis. Furthermore, IPB maintenance requires psychological support for ‘threat awareness’.

This study is the first to investigate the effect of practicums during the COVID-19 crisis on IPB in nursing students. Although it is a highly novel topic, there are limitations to this study. First, it has limited generalizability because the participants were third-year students from one university. This situation may become a confounding factor of student awareness. The study participants had similar years of study and university curriculums. Therefore, there is a need for future studies with a larger number of participants in different years.

In addition, because the imminent circumstances of the COVID-19 crisis surrounded this study, we could not verify the reliability and validity of the questions and scales used in the interviews.

We plan to refine the questionnaire’s content from this study, verify the reliability and validity of the questions and scales, and conduct a large-scale study to develop an infection-prevention program for safe practice.

## 5. Conclusions

In this study, we used a mixed-method approach to investigate the effect of nursing student practicums during the COVID-19 crisis on infection-prevention behavior. Our results showed that students used <Acquisition of knowledge and skills in IPMs during nursing practice> for <Defining the experience in infection-prevention measures>. Creating a new awareness about infection prevention led to <Changes in infection prevention behavior>. Therefore, for infection-prevention behavior to continue, there is a need to formulate prevention education in practice from real-practice experience.

## Figures and Tables

**Table 1:** Categories relating to nursing student awareness of IPMs for practicums during the COVID-19 crisis.

Category	Subcategory	Code
Acquisition of knowledge and skills in IPMs during nursing practice	Re-confirmation of hand hygiene methods	Confirmation of students’ knowledge of hand sanitizing by the practicum supervisor
		Learning about clinical infection control (including hand hygiene) from a nurse certified in infection control
	Learning about the correct usage of PPE	Skill acquisition for correct use and removal of masks, goggles, face shields, and aprons
		Learning how to care for patients with fever from the perspective of a nurse wearing PPE
	Minimizing patient contact time by shortening care time	Noticing that shortening care time takes precedence over infection control
		Learning about the difference in time required for acquiring nursing skills in on-campus practice situations and clinical placement practicums
	Hospital bed management for infected patients	Learning about zoning
		Learning about the role of the infection control division and the human resources involved in infection management
		Learning about nurse placements for infection control as well as bed control measures
	Thorough IPM implementation through supervision and strict adherence	Filling out the health/behavior management chart starting from 2 weeks before practicums
		Increasing the frequency of glove changing and sanitizing during patient care
		Sharing information about infection control at conferences
		Cleanliness care from the perspective of cleanliness/lack of hygiene as instructed by a nurse
		Wearing a mask at lunchtime and keeping your distance from others
Defining the experience in IPMs	Motivation for becoming a nurse	Learning the joy of nursing from the reactions of patients
		Noticing the importance of practicing patient care in clinical placement practicums
		From nursing practicums, aiming to be a nurse that patients need
	Gratitude and respect for patients/medical professionals	Tension from biases towards medical professionals as presented in the media
		The feeling of gratitude for being able to partake in practicums despite the COVID-19 crisis
Changes in attitude towards infection	Anxiety about infection before practicums	Student anxiety about infecting patients, medical professionals, or family
		Anxiety about infection when traveling by train to the clinical placement practicums
		Anxiety about lack of PPE at practicum facilities
		Anxiety about the discontinuation of the practicum or grades if a student gets infected
	The expectation for learning during practicums	Eagerness to learn during clinical placement practicums
		Encouragement from parents to learn during clinical placement practicums
		The expectation of learning from the first on-campus practice session
	Relief and a feeling of achievement after safe completion of the practicums	Not getting sick so breathing easy
		Reassurance that there were no infections among patients or medical professionals and no students who infected medical professionals
		A feeling of achievement after safe completion of the practicums
Changes in IPB	Thinking about IPM from the perspective of daily routines	Heightened awareness of health management from filling out the health/behavior management chart
		Motivation for IPM adherence from the pre-practicum and practicum orientation
		Making handwashing and mask-wearing habits
		Gathering information relating to infections from reliable information sources
		Reducing the number of people one goes out with
		Changing from train to bicycle transport to get to campus
	IPM for family	Reducing outings and eating out times with family
		Reducing opportunities to have contact with grandparents
		Making changes to part-time work
IPM-related issues	Stress due to IPM	Not being able to get a change in scenery after practicums due to outing limitations
		Hand irritation due to frequent handwashing and sanitizing
		The difficulty of use due to goggles fogging up or slipping off
		Closeness when communicating with patients with hearing difficulties
	Hesitancy towards health consultations if a fever develops	Keeping things to oneself
		Hesitancy in terms of consulting with others
		A feeling of guilt
		Concern about the effect on grades
	Lack of knowledge and skills from basic subjects	Lack of knowledge of microbiology and infectious disease medicine
		Lack of knowledge of hand sanitizer or disinfectant drugs

COVID-19, Coronavirus disease; IPB, infection-prevention behavior; IPM, infection-prevention measures; PPE, personal protective equipment.

**Table 2:** Participant Attributes.

Attribute		Meanor Number (*n* = 81)	SD or %
Age		21.3	0.7
Gender	Male	12	14.8
	Female	66	81.5
	Others	3	3.7
Fever of 37.5 °C or higher during the practicum period	Yes	6	7.4
	No	75	92.6
Field of participation on all the days of the clinical placement practicums	Adult nursing	68	84.0
	Gerontological nursing	16	19.8
	Women’s health nursing	12	14.8
	Community health nursing	12	14.8
	Child health nursing	6	7.4
	Psychiatric and mental health nursing	0	0
Frequency of clinical placement practicums (No. of fields)		2.3	3.0
Feelings towards clinical placement practicums	Want to go	40	49.4
	Do not want to go	12	14.8
	No choice	41	50.6

**Table 3:** Overview of pre-practicum orientations.

IPM-Related Topics from Pre-Practicum Orientations		No. of People	%
Participation in pre-practicum orientations	Less than 3 sessions	34	42.0
	3 or more sessions	47	58.0
Orientation topics that were helpful for IPMs	Standard precautions	63	77.8
	Infection route	61	75.3
	Timing of hand sanitizing	58	71.6
	Cleanliness/Lack of hygiene	42	51.9
	Chain of Infection	40	49.4
	A limited movement to cluster areas	40	49.4
	Avoid the three Cs	32	39.5
	Correct usage of PPE	24	29.6
	Virus incubation period	23	28.4
	Virus survival period	20	24.7

Standard precautions: These are prevention measures that can be universally applied during patient care regardless of infection status. Chain of Infection: These are the elements necessary for an infection to become established (pathogenic microorganism, an infected host, a portal of exit, infection route, a portal of entry, and a susceptible host [8]). Avoid the three Cs [9]: Crowded places, Close-contact settings, and Confined and enclosed spaces. PPE: masks and goggles, face shields, gloves, aprons, and gowns. We provided details on mask-wearing at lunchtime and eating without talking to the students. IPM, infection-prevention measure; PPE, personal protective equipment.

**Table 4:** Changes in anxiety factors before and after practicums.

Anxiety Factor	Before Practicums (*n* = 81)		After Practicums (*n* = 81)		*p*
	*n*	%	*n*	%	
A student infecting someone else	54	66.7	45	55.6	<0.01
A student being infected by someone else	54	66.7	45	55.6	<0.01
Train travel	42	51.9	22	27.2	<0.01
Cancellation of the practicum due to infection	41	50.6	10	12.3	<0.01
Lack of nursing skills	33	40.7	16	19.8	<0.01
The possibility of fever	26	32.1	25	30.9	<0.01
Lack of knowledge of infection control	8	9.9	5	6.2	<0.01

**Table 5:** A comparison of infection control measures learned by students from a clinical nurse and a nursing lecturer.

Item	Clinical Nurse (*n* = 81)		Nursing Lecturer (*n* = 81)		*p*
	*n*	%	*n*	%	
Correct usage of PPE	60	74.1	54	66.7	<0.01
Hand Hygiene	59	72.8	48	59.3	<0.01
Standard precautions	52	64.2	51	63.0	<0.01
Infection risk factors	50	61.7	43	53.1	<0.01
Nursing skills with consideration for infection prevention	46	56.8	35	43.2	<0.01
Distancing from patients	35	42.3	30	37.0	<0.01
Infection prevention at lunchtime	33	40.7	45	55.6	<0.01
Strict adherence to IPM	23	28.4	19	23.5	<0.01
Shortening care time	17	21.0	23	28.4	<0.01
The role of the infection control manager	16	19.8	10	12.3	<0.01

Hand Hygiene: Refers to washing hands under running water and hand sanitizing. Infection prevention at lunchtime: Refers to eating without talking and wearing a surgical mask immediately after eating. Nursing skills: Refers to the skills stipulated by the Ministry of Health, Labour and Welfare [10], including environmental adjustment skills, toileting assistance skills, hygiene/clothing, lifestyle assistance skills, skills for pain relief, maintaining comfort, meal assistance skills, activity/rest assistance skills, and skills for management of symptoms/bodily functions.IPM, infection-prevention measure; PPE, personal protective equipment.

**Table 6:** A comparison of IPBs during and after the practicum.

Item	During the Practicum (*n* = 81)		After the Practicum (*n* = 81)		*p*
	*n*	%	*n*	%	
Hand hygiene	79	97.5	76	93.8	<0.01
Mask wearing	79	97.5	74	91.4	<0.01
Avoiding outings	68	84.0	56	69.1	<0.01
Avoiding eating out	64	79.0	55	67.9	<0.01
Avoiding the three Cs	60	74.1	56	69.1	<0.05
Improving daily routines (meals and exercise)	17	21.0	15	18.5	<0.01
Infection prevention at lunchtime	43	53.1	21	25.9	<0.01

IPB, infection-prevention behavior.

**Table 7:** A comparison of IPBs that were continued after the practicums, according to the number of pre-practicum orientations attended and experience in clinical placement practicums.

Behavior that Continued after the Practicums	Participation in Orientation				*p*	Experience in Clinical Placement Practicums				*p*
	Less than 3 Sessions (*n* = 34)		3 or more Sessions (*n* = 47)			Less than 3 Sessions (*n* = 64)		3 or more Sessions (*n* = 17)		
	*n*	%	*n*	%		*n*	%	*n*	%	
Hand Hygiene	28	82.4	42	89.4	0.36	59	92.2	11	64.7	<0.01
Avoiding the three Cs	19	55.9	37	78.7	<0.05	45	70.3	11	64.7	0.66
Infection prevention at lunchtime	19	55.9	37	78.7	<0.05	16	25.0	5	29.4	0.71

IPBs, infection-prevention behaviors.

## Data Availability

The study data are available on request from the corresponding author.
